# Engineering Circulating Tumor Cells as Novel Cancer Theranostics

**DOI:** 10.7150/thno.44259

**Published:** 2020-06-29

**Authors:** Katie M Parkins, Veronica P Dubois, John J Kelly, Yuanxin Chen, Natasha N Knier, Paula J Foster, John A Ronald

**Affiliations:** 1 Robarts Research Institute, The University of Western Ontario, London, Ontario, Canada.; 2 The Department of Medical Biophysics, The University of Western Ontario, London, Ontario, Canada.; 3 Lawson Health Research Institute, London, Ontario, Canada.

**Keywords:** self-homing, CTC, self-targeted therapy, drug delivery, metastasis

## Abstract

New ways to target and treat metastatic disease are urgently needed. Tumor “self-homing” describes the recruitment of circulating tumor cells (CTCs) back to a previously excised primary tumor location, contributing to tumor recurrence, as well as their migration to established metastatic lesions. Recently, self-homing CTCs have been exploited as delivery vehicles for anti-cancer therapeutics in preclinical primary tumor models. However, the ability of CTCs to self-home and treat metastatic disease is largely unknown.

**Methods:** Here, we used bioluminescence imaging (BLI) to explore whether systemically administered CTCs home to metastatic lesions and if CTCs armed with both a reporter gene and a cytotoxic prodrug gene therapy can be used to visualize and treat metastatic disease.

**Results:** BLI performed over time revealed a remarkable ability of CTCs to home to and treat tumors throughout the body. Excitingly, metastatic tumor burden in mice that received therapeutic CTCs was lower compared to mice receiving control CTCs.

**Conclusion:** This study demonstrates the noteworthy ability of experimental CTCs to home to disseminated breast cancer lesions. Moreover, by incorporating a prodrug gene therapy system into our self-homing CTCs, we show exciting progress towards effective and targeted delivery of gene-based therapeutics to treat both primary and metastatic lesions.

## Introduction

Cancer patient outcomes have significantly improved in the last few decades due to superior imaging, surgical, and radiotherapy techniques, the recent application of 'omics' lesion profiling to guide therapy, and more efficacious drugs 1. These advances now allow many patients with localized primary tumors or minimal metastatic disease at the time of diagnosis to be effectively managed. Despite these transformative advances, the ability to benefit patients with highly disseminated metastatic disease remains a significant challenge. Difficulties with controlling metastatic disease include, but are not limited to, the lack of tools to visualize lesions at an earlier stage when they may be more readily treated, insufficient systemic delivery of therapeutics to all lesions, and, most notably, extensive tumor heterogeneity both within and between lesions throughout the body 2-4. These substantial barriers highlight an unmet need for new technologies to effectively visualize and treat metastatic lesions, preferably so-called theranostic tools that have both diagnostic and therapeutic capabilities.

Cells are an attractive form of theranostic vector as they can be readily engineered *ex vivo* prior to transplantation with both imaging reporter genes for noninvasive localization and therapeutic transgenes 5-8. While some cell types have been shown to naturally home to lesions such as stem cells and immune cells 9-15, one can also engineer cells with receptors targeting tumor-associated antigens to redirect *in vivo* cellular tropism. Recently, chimeric antigen receptor T cells (CAR-T cells) targeting the B cell antigen CD-19 became the first genetically-modified cell-based therapies to be approved for patients with relapsed or refractory B-cell precursor acute lymphoblastic leukemia and large B cell lymphoma 16-19. While substantial efforts are now aimed at using CAR-T cells for the treatment of solid tumors, so far, their less than ideal therapeutic effectiveness has been attributed to insufficient tumor-homing and/or intratumoral immunological barriers 20. Thus, the continued exploration of alternative cell types that can effectively home to metastatic solid tumors for use as novel theranostic vectors is warranted.

Paget's “seed and soil hypothesis” describes the wide dissemination of “seeds”, or circulating tumor cells (CTCs), from a primary tumor and the formation of overt metastases selectively in “soils” that permit CTC survival and proliferation 21. However, due to the non-permissive nature of tumor-free organs, metastasis has been shown to be an inefficient process in both experimental animal models and cancer patients 22-24. The impedance of the formation of new metastases has been partly attributed to both vascular barriers that inhibit CTC extravasation from the blood as well as unfavorable survival conditions 25. Conversely, shed CTCs have been shown to be highly capable of homing back to their tumor of origin, a concept termed tumor “self-seeding” that was first suggested and demonstrated by Norton and Massague 26. Self-seeding has been shown in animal models of human breast, colon and melanoma cancer, and is theorized to contribute to tumor recurrence following resection 27. Unlike in tumor-free organs, tumor vasculature is often “leaky” due to a compromised vascular endothelium, and thus, more easily facilitates the extravasation of CTCs back into their original tumors 28-29. Moreover, the primary tumor microenvironment is considered highly permissive soil for the continued survival and growth of recruited CTCs, leading to the expansion of highly metastatic clones that have a higher capacity to seed distant organs 27. Similarly, metastatic lesions that have formed in distant organs are also considered fertile soil for additional “self-homing” CTCs to migrate to, survive, and expand within, which may contribute to accelerated metastatic disease progression 27.

In the last two decades, several groups have exploited self-homing CTCs as “self-targeted” delivery vehicles for *ex vivo* loaded anti-cancer therapeutic cargo 30-35. Cargo has included oncolytic viruses such as the H-1 parvovirus and vesicular stomatitis virus (VSV), prodrug converting enzyme genes including herpes simplex virus thymidine kinase (HSV-TK) and cytosine deaminase (CD), transgenes that target the tumor microenvironment such as tumor necrosis factor (TNF), and the secretory version of TNF-related apoptosis-inducing ligand (S-TRAIL). Additionally, a few groups have co-engineered the therapeutic CTCs and/or their viral cargo with optical or positron emission tomography (PET) imaging reporter genes to enable the fate of the cells/cargo to be noninvasively monitored with reporter gene imaging 31-33, 35. Importantly, while the ability to target, visualize, and treat singular pre-established subcutaneous tumors as well as orthotopic or metastatic lesions in a singular organ (e.g., lungs 31 or brain 35) has been demonstrated, to the best of our knowledge, the ability of self-homing CTCs to migrate into and be used to visualize and treat spontaneous multi-organ metastatic disease is largely unknown.

Here, we employed longitudinal reporter gene imaging to show that systemically administered engineered CTCs efficiently home to both orthotopic and spontaneous metastatic breast cancer lesions. Both CTCs and metastatic cancer cells were engineered with orthogonal luciferase transgenes, allowing for dual bioluminescence imaging (BLI). Further, we demonstrate that CTCs armed with both a BLI reporter and the gene for the prodrug converting enzyme cytosine deaminase-uracil phosphoribosyltransferase (CD:UPRT) can be used to visualize and treat metastatic disease. Our preclinical study supports engineered CTCs as a novel self-targeting cellular theranostic platform for the visualization and treatment of distributed metastases - the most relevant lesions to patient outcome.

## Results

### Tracking of Self-Homing Cancer Cells in a Contralateral Orthotopic Tumor Model

Previous studies have shown that breast cancer cells from one mammary fat pad (MFP) can home to a contralateral MFP tumor 27. Thus, we first started exploring the use of dual-BLI to monitor tumor self-homing using this same experimental setup. We engineered the mouse breast cancer cell line (4T1) and its brain-seeking metastatic variant (4T1BR5) to express the orthogonal BLI reporters *Renilla* luciferase (RLuc) and *Firefly* luciferase (FLuc), respectively. This allowed us to sensitively track both populations in the same animal over time. 4T1 cells were transduced with a lentiviral vector encoding both RLuc and ZsGreen and sorted to obtain 4T1-RLuc cells (Figure S1A). No significant change in ZsGreen expression over multiple passages was observed (Figure S1B) and there was a significant positive correlation shown between the number of 4T1-RLuc cells and RLuc/ZsGreen signal (R^2^ = 0.99, *p<0.001*; Figure S1C). FLuc-expressing 4T1BR5 (4T1BR5-FLuc) cells were engineered and characterized similarly in a previous study 36. We next ensured a lack of cross-reactivity of the luciferase substrates. 4T1BR5-FLuc cells incubated with the FLuc substrate D-luciferin demonstrated significantly higher BLI signal than 4T1-RLuc cells, 4T1 parental cells, or equivalent volume of media *(p<0.001*; Figure S1D). 4T1-RLuc cells also did not produce signal significantly different than 4T1 parental cells or media alone. Similarly, after the addition of RLuc substrate h-Coelenterazine, 4T1-RLuc cells had significantly higher BLI signal than 4T1BR5-FLuc cells, 4T1 parental cells, or equivalent volume of media *(p<0.001*; Figure S1E*)*. As expected, no significant differences in BLI signal were seen between 4T1BR5-FLuc cells, 4T1 parental cells or media alone. We next explored the migration of our engineered cells towards conditioned media from both cell lines using transwell migration assays. A significant increase in cell migration was seen for 4T1BR5-FLuc cells when conditioned media from 4T1-RLuc cells was used compared to conditioned media from 4T1BR5-FLuc cells or unconditioned media *(p<0.01*; Figure S1F*).* A significant increase in cell migration was also seen for 4T1-RLuc cells when conditioned media from 4T1-RLuc cells was used compared to unconditioned media *(p<0.01*; Figure S1F*).*

**Figure 1:**
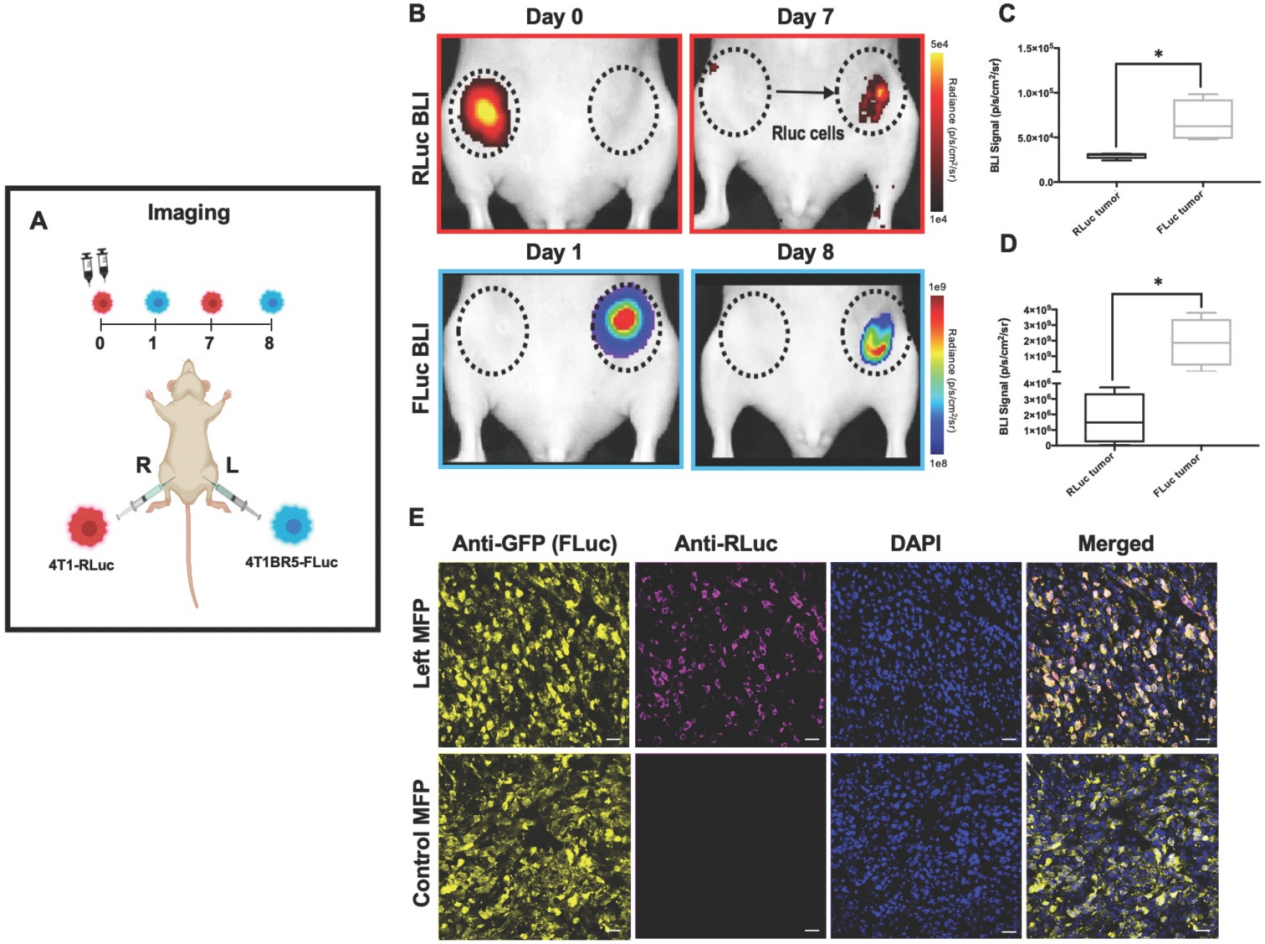
** Experimental timeline for contralateral tumor self-homing model** (n=5) (**A**). On Day 0 after cell injection, 4T1-RLuc cells only showed signal after administration with h-coelenterazine and on Day 1, 4T1BR5-FLuc cells only showed signal after administration of D-Luciferin. By day 7, 4T1BR5-FLuc cells did not appear to migrate as FLuc signal was not detected in the contralateral MFP but 4T1-RLuc cells could be detected in the contralateral MFP tumor (**B**). RLuc signal on day 7 was significantly higher in the contralateral MFP compared to the ipsilateral MFP on day 8 (**C-D**). The presence of both 4T1-RLuc and 4T1BR5-FLuc cells in the left MFP was confirmed histologically and compared to a control MFP tumor composed of only FLuc expressing cells (scale bars=200 microns) (**E**).

4T1-RLuc cells were then implanted into the right MFP of nude mice (n=5) and 4T1BR5-FLuc cells were implanted into the contralateral (left) MFP (Figure 1A). This allowed us to validate the lack of substrate cross-reactivity *in vivo* at early time points after cell injection (Days 0 and 1; Figure 1 and Figure S2) as well as evaluate whether either of the cell lines migrated into the contralateral MFP tumor (Figure 1). On Day 0 after cell injection, 4T1-RLuc cells only showed signal after administration with h-coelenterazine and on Day 1, 4T1BR5-FLuc cells only showed signal after administration of D-luciferin (Figure S2). By day 8, 4T1BR5-FLuc cells did not appear to migrate as FLuc signal was not detected in the contralateral MFP and was significantly higher in the ipsilateral MFP (*p<0.05*; Figure 1D). In contrast, 4T1-RLuc cells could be detected in the contralateral MFP tumor, and RLuc signal was significantly higher in the contralateral compared to ipsilateral MFP (*p<0.05*; Figure 1C). The presence of both 4T1-RLuc and 4T1BR5-FLuc cells in the left MFP was confirmed histologically (Figure 1E), supporting our BLI results and validating that the 4T1-RLuc cells migrated to the contralateral MFP tumor.

### Primary Tumors and Spontaneous Metastases can be Visualized with Systemically Administered “Diagnostic” CTCs

**Figure 2:**
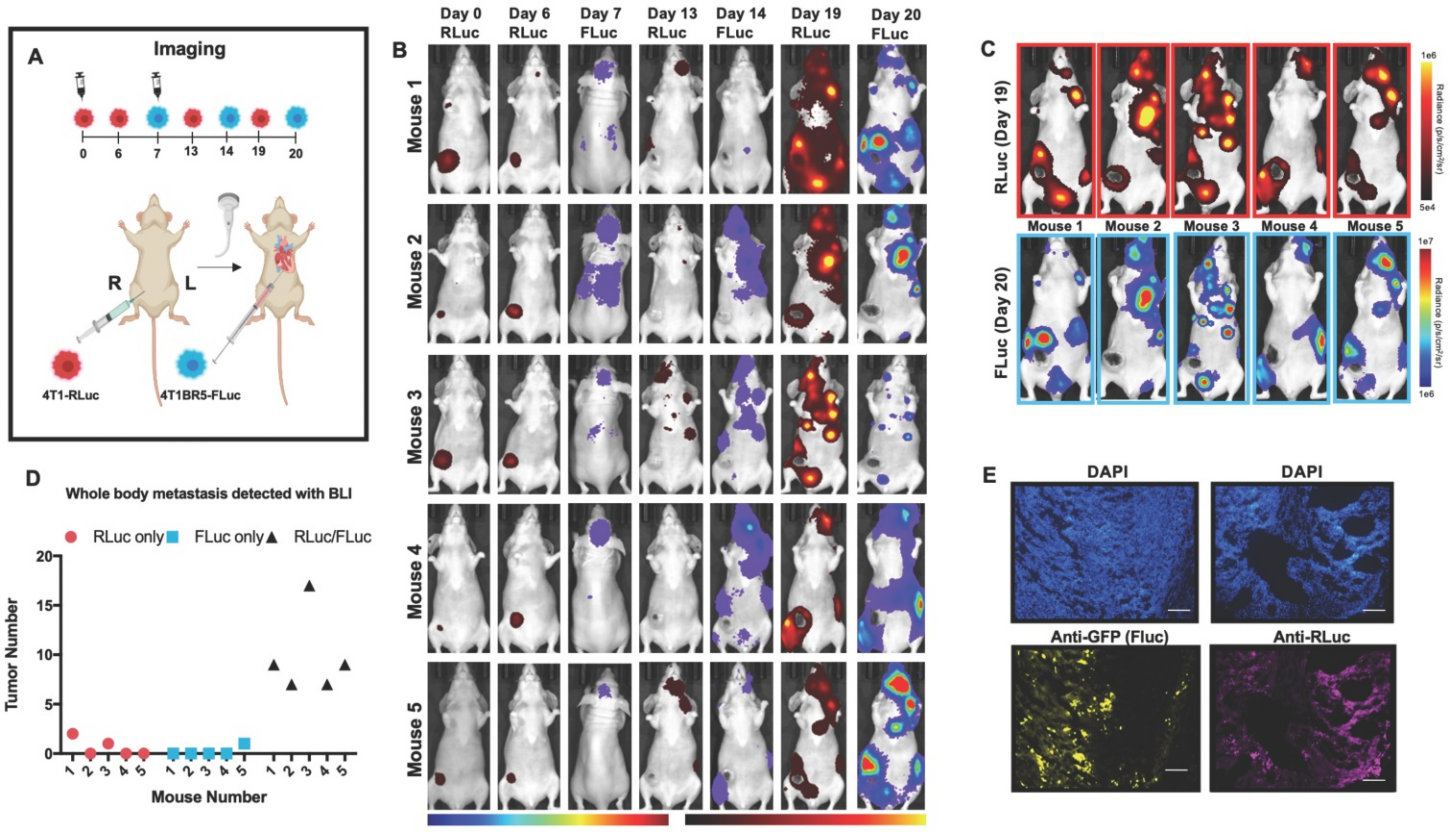
** Experimental timeline for visualizing diagnostic CTCs** (n=5) (**A**). RLuc BLI was performed on days 0, 6, 13 and 19 to visualize cells in the right MFP and any spontaneous metastases and FLuc BLI was performed on days 7, 14 and 20 to visualize CTCs; these images have not been scaled to enable the visualization of all lesions at each individual time point (**B**). FLuc-expressing CTCs efficiently homed to RLuc-expressing primary tumors and spontaneous metastases throughout the body (**C**). Quantitative analysis of endpoint BLI images (day 19 and 20) revealed that the vast majority of metastases were composed of both 4T1-RLuc and 4T1BR5-FLuc cells, which was significantly higher than the number of metastases that were either 4T1-RLuc-positive only or 4T1BR5-FLuc-positive only (*p< 0.001*) (**C-D**). The presence of both 4T1-RLuc and 4T1BR5-FLuc cells in a brain metastasis was confirmed histologically (scale bars= 500 microns) (**E**).

We next assessed the ability of systemically administered CTCs to home to primary tumors and spontaneous metastases. We implanted 4T1-RLuc cells into the right MFP of nude mice (n=5) and allowed tumors to grow for 7 days prior to injecting 4T1BR5-FLuc CTCs via an intracardiac injection under ultrasound guidance (Figure 2A). RLuc BLI was performed on days 0, 6, 13 and 19 to visualize cells in the right MFP and any spontaneous metastases and FLuc BLI was performed on days 7, 14 and 20 to visualize CTCs (Figure 2A). RLuc BLI showed the presence of metastases in 1 of 5 mice on day 6 prior to CTC injection. RLuc tumors were often found in the brain and/or hind limbs. FLuc BLI over time revealed the ability of FLuc-expressing CTCs to home to RLuc-expressing primary tumors and spontaneous metastases throughout the body (Figure 2B). Quantitative analysis of endpoint BLI images (day 19 and 20) revealed that the vast majority of metastases were composed of both 4T1-RLuc and 4T1BR5-FLuc cells (9.8±1.9), which was significantly higher than the number of metastases that were either 4T1-RLuc-positive only (0.6±0.4; *p<0.01*) or 4T1BR5-FLuc-positive only (0.2±0.2; *p<0.001*) (Figure 2C-D, Figure S3). The presence of both 4T1-RLuc and 4T1BR5-FLuc cells in numerous metastases was confirmed histologically (Figure 2E, Figure S3B), supporting our BLI results.

### Self-Seeding “Theranostic” CTCs Can Migrate into and Treat Primary Tumors and Spontaneous Metastases

**Figure 3:**
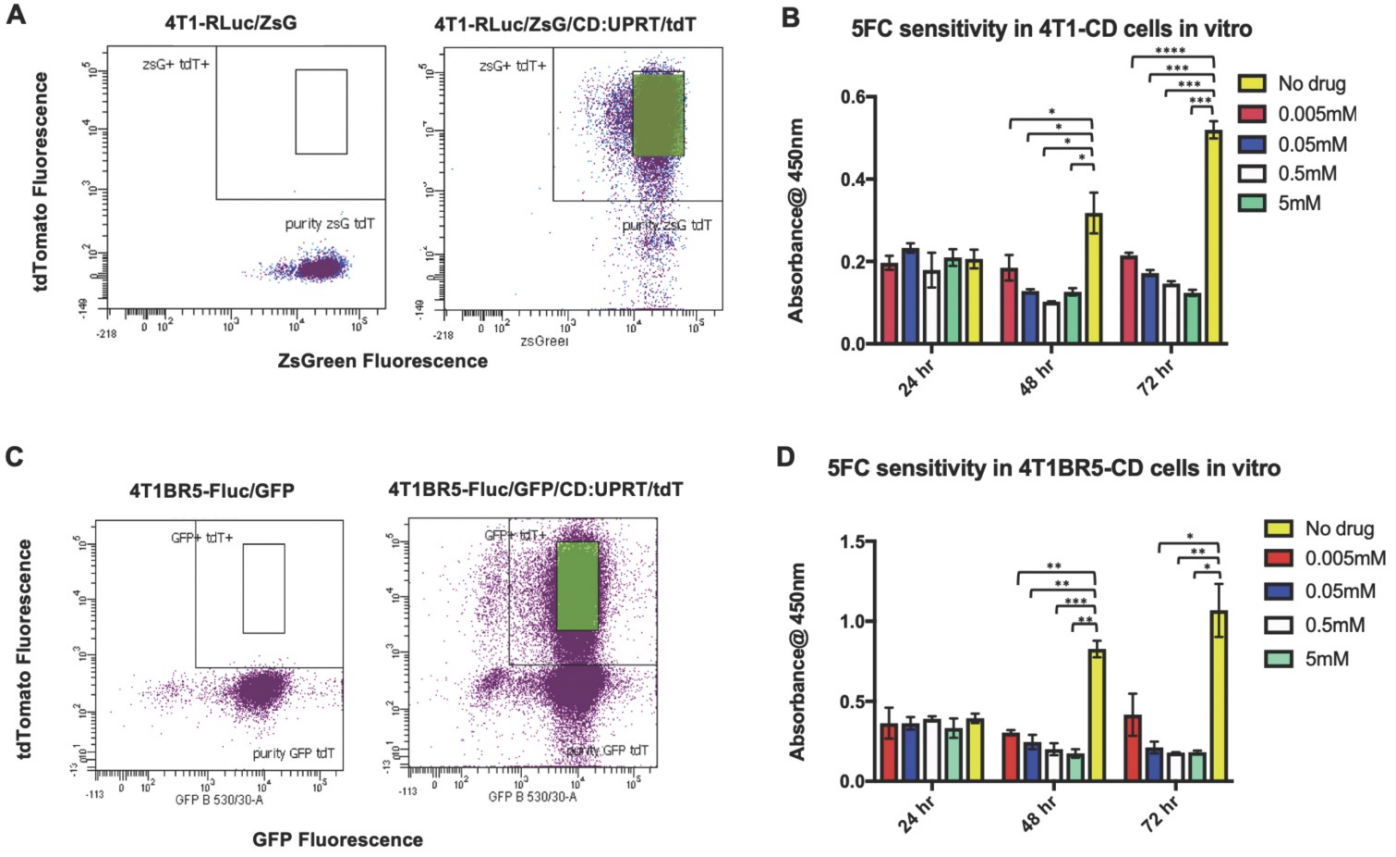
4T1-RLuc and 4T1BR5-FLuc cells were transduced with a lentiviral vector co-expressing the therapeutic prodrug converting fusion enzyme cytosine deaminase-uracil phosphoribosyltransferase (CD:UPRT) and tdTomato (tdT), and sorted via tdT to obtain 4T1-RLuc/CD (**A**) and 4T1BR5-FLuc/CD cells (**C**). After 72 h of incubation with 5'FC, CD expressing cells showed significantly less survival than cells without drug (**B-D**).

We next investigated whether self-homing CTCs expressing the therapeutic prodrug converting fusion enzyme cytosine deaminase-uracil phosphoribosyltransferase (CD:UPRT) could be systemically administered to treat primary and disseminated lesions. CD:UPRT converts non-toxic 5'-fluorocytosine (5'FC) into the cytotoxic compound 5'fluoruridine monophosphate (5'FUMP) and was chosen as a suicide switch to eliminate the therapeutic cells as well as a way to kill adjacent non-engineered cancer cells via the bystander effect 37-40. 4T1-RLuc and 4T1BR5-FLuc cells were transduced with a lentiviral vector co-expressing CD:UPRT (CD for brevity) and tdTomato (tdT), and sorted via tdT to obtain 4T1-RLuc/CD (4T1-CD) cells and 4T1BR5-FLuc/CD (4T1BR5-CD) cells (Figure 3A, C). After 72 h of incubation with 5'FC (0.005 mM, p<0.0001; 0.05 mM, 0.5 mM, 5 mM, p<0.001), 4T1-CD cells showed significantly less survival than cells without drug (Figure 3B). Similarly, after 72 h of incubation with 5'FC (0.05 mM, 5 mM, p<0.05; 0.5, p<0.01), 4T1BR5-CD cells showed significantly less survival than cells without drug (Figure 3D).

**Figure 4:**
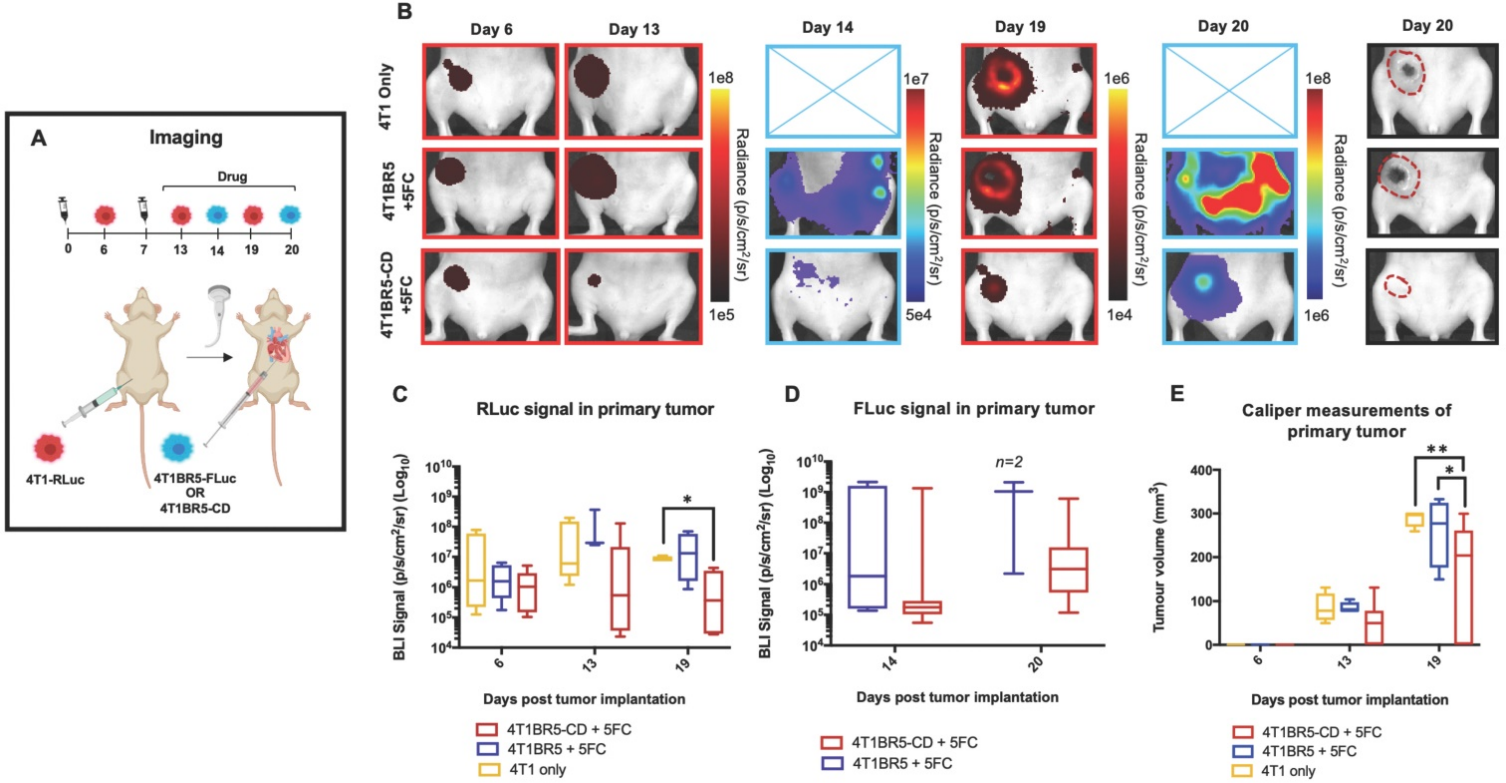
** Experimental timeline for visualizing self-homing theranostic CTCs** (n=15) (**A**). On day 6 (prior to drug administration), mice receiving 4T1BR5-CD cells had MFP RLuc signal that was not significantly different than mice receiving 4T1BR5 cells or 4T1 cells only (**B**). By day 19, mice receiving only 4T1-RLuc cells had significantly higher RLuc signal in the MFP compared to mice that received 4T1BR5-CD cells (**C**). On day 14, there was not a significant difference observed in FLuc signal between mice that received 4T1BR5-CD cells or 4T1BR5 cells (**D**). At endpoint, primary tumor burden measured by calipers was significantly higher in mice that received only 4T1 cells compared to mice that received 4T1BR5-CD cells (**E**).

**Figure 5:**
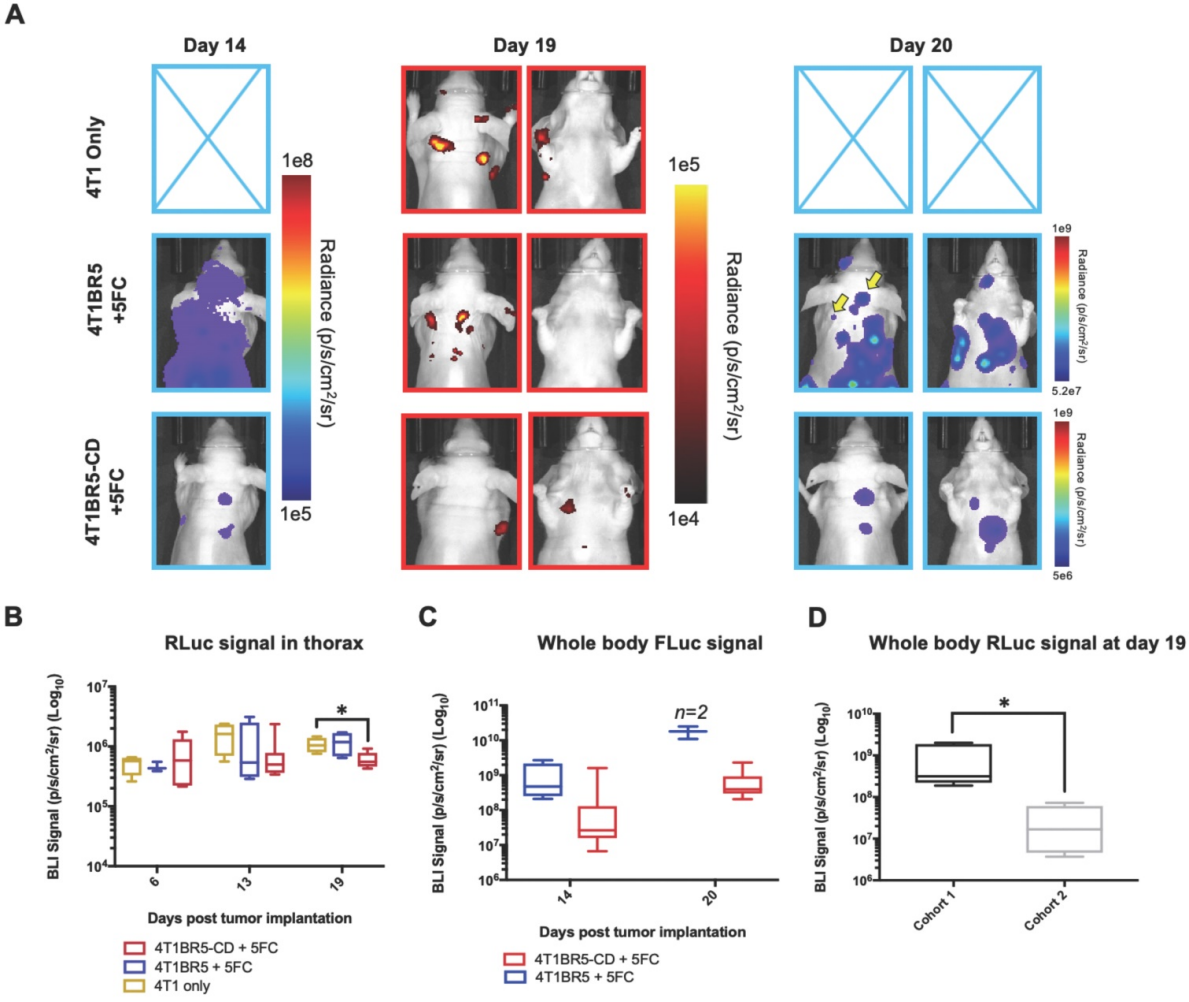
** Treating spontaneous metastases with self-homing theranostic CTCs:** Spontaneous metastases were visualized with RLuc BLI on day 19 and CTCs with FLuc BLI on days 14 and 20 (**A**). Mice receiving 4T1BR5-CD cells had significantly lower metastatic burden than 4T1 only mice at day 19 (**A-B**). In the two control mice (4T1BR5-Fluc) that reached endpoint, FLuc CTCs were visualized in the same location as RLucexpressing spontaneous metastases (yellow arrows) as well as at new sites throughout the body (A). We hypothesized that the reduced overlap in RLuc and FLuc signals in these experiments may be due to less RLuc tumor burden in this latter cohort of mice compared to mice in the previous experiment shown in Figure 2, leading to less established sites for the CTCs to home. Comparing the RLuc signal between the two cohorts of mice revealed a significantly lower signal in the latter group (**D**).

Finally, 4T1-RLuc cells were implanted into the right MFP of nude mice and allowed to grow and metastasize for 7 days prior to intracardiac injection of either 4T1BR5-CD cells (n=7) or 4T1BR5-FLuc cells (n=4), or mice did not receive any cells intracardially (4T1 only; n=4) (Figure 4A). Mice receiving cells were then treated with 5'FC daily from days 10 to 20. RLuc BLI was performed on all mice on days 6, 13 and 19 to visualize cells in the primary tumor and any spontaneous metastases, and FLuc BLI was performed on days 14 and 20 to visualize CTCs (Figure 4A). The effects of treatment on primary tumors and metastatic disease were assessed separately (Figures 4-5). On days 6 (prior to drug administration), and 13 (3 days post drug administration), mice receiving 4T1BR5-CD cells had primary tumor signal that was not significantly different than both control groups (Figure 4B-C). However, by day 19, mice receiving 4T1BR5-CD cells had significantly lower primary tumor RLuc signal than mice receiving 4T1 cells only (*p<0.05*; Figure 4B-C). At day 14, 4 days after 5'FC treatment was initiated, FLuc signal between the groups was not significantly different from each other (Figure 4B-D). While we observed a qualitative difference in FLuc signal in the primary tumor at day 20 (Figure 4B), 2/4 mice that received 4T1BR5-FLuc cells had to be sacrificed prior to endpoint due to both the size of the tumors and presence of ulceration and thus, a statistical test could not be performed at this time point (Figure 4D). At endpoint (day 20), primary tumors were not palpable in 3/7 mice that received 4T1BR5-CD expressing cells compared to all mice in the control groups having palpable primary tumors. In addition, 4T1BR5-CD mice had significantly lower primary tumor volumes compared to 4T1 only mice (p<0.01) and mice that received 4T1BR5-FLuc cells (*p<0.05*) (Figure 4B-E). Mice without palpable tumors at endpoint were considered “full responders” and noted with an asterisk on individual mouse response curves (Figure S6). Of the remaining 4 mice that received 4T1BR5-CD cells, 3 were considered “partial responders” (tumor burden < 250mm^3^), and 1, “non-responder” (tumor burden > 250mm^3^) compared to 7 of 8 control mice that were considered “non-responders” and one “partial responder”. To assess metastatic burden separately from primary tumor burden, regions of interest were drawn over the upper half of each mouse and RLuc BLI average radiance measurements from both the dorsal and ventral sides of each mouse were added together. This analysis showed that mice receiving 4T1BR5-CD cells had significantly lower metastatic burden than 4T1 only mice at day 19 (*p<0.05*; Figure 5A-B). In the 2 control mice (4T1BR5-Fluc) that reached endpoint, FLuc CTCs were visualized in the same location as RLuc-expressing spontaneous metastases as well as at new sites throughout the body (Figure 5A-C). We hypothesized that the reduced overlap in RLuc and FLuc signals in these experiments may be due to less RLuc tumor burden in this latter cohort of mice compared to mice in the previous experiment shown in Figure 2, leading to less established sites for the CTCs to home. Comparing the RLuc signal between the two cohorts of mice revealed a significantly lower signal in the latter group (*p<0.05*; Figure 5D). Overall, our data support the use of systemically administered “theranostic” CTCs to treat primary tumors, and with further development could be used to treat metastatic tumors.

## Discussion

Cancer, particularly in patients with metastatic disease, remains a leading cause of death in the world 41, 42. Treatments that often work on localized disease are often not an option or fail in the patients with significant metastatic spread. Thus, the development of technologies for earlier detection and treatment of metastatic disease remains at the forefront of cancer research. This study demonstrates that engineered “self-homing” CTCs co-expressing an imaging reporter and a therapeutic transgene can be used as a novel “theranostic” cellular vector to visualize and treat both primary tumors and disseminated spontaneous breast cancer metastases in mice. We first show, using dual-luciferase BLI, the ability of systemically administered CTCs to preferentially home to pre-established spontaneous metastases in various organs throughout the body. Leveraging on this highly preferential homing capability, we then show that CTCs co-expressing the prodrug converting fusion enzyme system CD:UPRT can kill themselves via a suicide switch as well as neighboring cells via the bystander effect to decrease tumor burden.

Tumor “self-homing” describes the recruitment of CTCs back to an original tumor site, contributing to tumor recurrence, as well as accelerated primary tumor growth. Self-homing has been largely attributed to an established tumor microenvironment that is considered highly permissive soil for the survival and growth of recruited CTCs 26. Numerous studies have shown self-homing of systemically administered CTCs to an established primary tumor or singular metastatic lesion 27, 30-35. Importantly, our study here demonstrates that CTCs administered intracardially are also efficient at homing to metastases that have spontaneously disseminated throughout the body (Figure 2). In our model, primary tumors had a week to grow and spontaneously metastasize throughout the body prior to the injection of experimental CTCs. Our imaging data from early time points supports the idea that CTCs homed to pre-established lesions rather than vice versa, as some animals displayed spontaneous metastases pre-CTC injection at the locations where CTCs homed to after intracardiac injection. Although we used a preclinical imaging reporter in these studies, we posit that, with significant effort to ensure safety, it might be possible to use highly-efficient self-homing CTCs engineered with clinically-relevant reporter genes (e.g., for PET or MRI) as a possible diagnostic technology for visualizing metastatic lesions in patients. A similar cell-based cancer diagnosis strategy was recently described, demonstrating the use of systemically-administered macrophages that were engineered to secrete a reporter gene that is detectable in the urine when they become “activated” (i.e., differentiated) within tumors 43.

Based on the efficiency of CTC self-homing to primary and metastatic lesions, we also explored and demonstrate their use as therapeutic vectors. In comparison to previously used cellular vectors (e.g., stem cells and immune cells), cancer cells may have some advantages as they can be continuously grown *in vitro* enabling extensive cell engineering, may have superior homing capability to lesions, and may survive longer and expand more once in tumors 44-57. In fact, several other groups have previously repurposed the self-seeding properties of cancer cells to use them as “self-targeted” vectors for anti-cancer therapeutics 30-35. However, the majority of these studies focused on the treatment of a singular primary or metastatic lesion. We engineered our experimental CTCs to express the suicide gene CD:UPRT and used dual BLI to monitor the effects of self-targeted therapy. By incorporating CD:UPRT and FLuc into CTCs, we were able to visualize with BLI that 5'FC administration was able to attenuate the growth of CTCs compared to mice that received FLuc-expressing CTCs lacking CD:UPRT (Figures 4 and 5). However, the self-killing of CTCs was not 100% effective as all mice showed residual FLuc signal at endpoint (Figures 4 and 5). Therefore, the safety of our first iteration of this strategy is less than ideal and needs further refinement. Importantly, we also visualized the CTC-mediated killing of adjacent RLuc-expressing cancer cells in both primary and metastatic lesions (Figures 4 and 5). However, again at endpoint there was still residual RLuc signal, particularly for metastatic lesions, and thus further refinement of this strategy is needed to optimize the treatment of established lesions.

One important aspect that may influence the ability to induce a significant therapeutic effect is the ratio of therapeutic CTCs to cancer cells in a particular lesion. For instance, in our contralateral model, we visualized that therapeutic CTCs had successfully migrated from the original to the contralateral mammary fat pad tumor, but upon 5'FC administration we did not observe a significant therapeutic effect (Figure S4). We hypothesized that perhaps the number of therapeutic CTCs that had homed to the contralateral tumor was too low to promote sufficient killing of adjacent cancer cells. In support of this theory, when 3×10^5^ RLuc-expressing CTCs were injected directly into the mammary fat pad tumor, this generated higher RLuc signal than when they naturally homed there, and this time significantly reduced tumor burden after 5'FC administration (Figure S5). We also saw evidence that the ratio of CTCs to tumor size was important in our metastatic models. Large primary tumors that could theoretically attract more CTCs were readily treated, with some primary tumors becoming unpalpable at endpoint. In contrast, in smaller metastatic lesions that may not be able to accrue as many CTCs as primary tumors, the therapeutic effects were less dramatic. It will be important to explore ways to further improve the efficiency of therapeutic CTC homing to smaller tumors to maximize delivery of therapeutic cargo. Current work is also looking at treating metastatic disease in mice where the primary tumor has been surgically removed prior to CTC administration so that the CTCs may respond to homing signals from smaller metastases more effectively. Additionally, the timing between CTC injection and 5'FC administration should be further explored. If we administer the pro-drug too early, we may lose some of our therapeutic CTCs to self-induced toxicity prior to receiving any therapeutic effects on neighboring non-engineered cancer cells. Our data suggests that three days may not be the optimal window as we were unable to completely eliminate metastatic lesions. A larger window may increase therapeutic efficacy by allowing therapeutic CTCs to better migrate into and expand within tumors in order to increase the ratio of CTCs to neighbouring pre-existing cancer cells prior to 5'FC administration.

The self-seeding capabilities of cancer cells have been attributed to both the recruitment potential of the established tumor microenvironment as well as the seeding capabilities of cancer cells themselves. While we have not evaluated mechanism in our study, previous studies have demonstrated cytokines IL-6 and IL-8 that are produced by aggressive tumor types, including breast carcinomas, can act as chemoattractants to efficiently recruit CTCs 27, 58-60. Kim et al., have also shown using the 4T1 model that recipient tumors that are seeded by CTCs show a significant increase in leukocyte recruitment compared to unseeded tumors, which can lead to increased production of IL-6. Additionally, Vilalta et al., have shown that irradiation of 4T1 tumors leads to increased production of granulocyte macrophage colony stimulating factor (GM-CSF) which also stimulates the recruitment of CTCs to a tumor 61. This work agrees with previous findings that have shown sublethal doses of irradiation can promote cancer cell migration and invasion 62-66. Thus, factors derived from both the stroma and the tumor may function together to attract migrating CTCs back to an established tumor site. Another factor that can affect how well cancer cells can efficiently home to an established tumor is their metastatic potential 27. Our transwell migration assay results showed that conditioned media from primary 4T1 cells caused increased migration compared to media from our experimental CTC 4T1BR5 cell line. However, in our contralateral tumor model, the 4T1BR5 cells grow faster than the 4T1 cells and thus, develop a palpable tumor earlier than the 4T1 cells. In this model, we visualized 4T1 cells that had migrated to the 4T1BR5 tumor which we hypothesized may be due to a more established tumor microenvironment.

While our findings suggest CTCs have potential as highly efficient carriers of therapeutic cargo to primary and metastatic tumor sites, our approach has some limitations to consider. Most importantly, CD:UPRT appeared less than ideal at killing both engineered CTCs and neighbouring non-engineered cancer cells. Future work will explore incorporating more than one therapeutic gene into the engineered cells. For example, a CD:UPRT/HSV-TK system, would allow the administration of two different pro-drugs, creating a higher likelihood of targeting and treating engineered CTCs while possibly also enhancing the bystander effect 67-72. Finally, BLI was used in the current work to enable highly sensitive and specific tracking of two different cell populations within the same animal. However, due to the aggressive nature of the 4T1 model, orthotopic tumors often ulcerate preventing some light from being collected by the charge-coupled device (CCD) camera 73-75. This may have had an effect on our endpoint imaging results in the orthotopic tumor model since untreated tumors were larger in size with more ulceration present, which is why we also performed caliper measurements of tumor volumes. Future studies should investigate alternative or complementary imaging tools that may more accurately quantitate tumor volumes around the body (e.g., MRI).

In conclusion, our work provides evidence that CTCs are a novel theranostic vector platform for the visualization and treatment of pre-established tumor sites throughout the body. Overall, while further refinement is needed, this unorthodox strategy may have tremendous long term translational potential as a highly effective theranostic, specifically in patient populations presenting with metastatic disease at initial diagnosis, and those at high risk of cancer recurrence or metastatic relapse.

## Materials and Methods

### Study Design

The primary research objective of this work was to visualize and treat disseminated disease with engineered self-homing CTCs in a mouse model of breast cancer metastasis. A minimum of three technical replicates were performed for all *in vitro* experiments and four to eight animals per group were used for *in vivo* experiments. For treatment studies, animals were randomized before imaging and treatment. All outliers were included in the analysis and no datasets were excluded. Authors were not blinded to the results.

### Lentiviral Production

To produce RLuc8/ZsG and CD:UPRT/tdT lentiviruses, third-generation packaging and envelope-expression plasmids acquired from Addgene [pMDLg/pRRE (#12251), pRSV-Rev (#12253), pMD2.G (#12259)] and lentiviral transfer vectors encoding the desired gene were co-transfected into human embryonic kidney (HEK 293T) cells using Lipofectamine 3000 according to the manufacturer's production protocol (Thermo Fisher Scientific Inc., USA). Lentivirus supernatants were harvested at 24- and 48-h post transfection, filtered through a 0.45μm filter and stored at -80°C prior to lentiviral transduction.

### Cell Engineering

The 4T1BR5 cells were a kind gift from Dr. Patricia Steeg's lab and transduced with a commercial lentiviral vector (RediFect Red-FLuc-GFP; PerkinElmer, USA). Cells were FACS sorted based on GFP expression using a FACSAria III flow cytometric cell sorter (BD Biosciences, USA). The parental 4T1 cells were also received from Dr. Patricia Steeg's lab and transduced with an in-house RLuc8/ZsG lentivirus. Cells were sorted based on ZsG expression using FACS. The resultant 4T1BR5-FLuc/GFP (4T1BR5-Fluc) and 4T1-RLuc/ZsG (4T1-Rluc) cells were maintained in DMEM containing 10% FBS and 1% antibiotics, at 37°C and 5% CO_2_, and then transduced a second time using an in-house CD:UPRT/tdT lentivirus. Both cell lines were FACS sorted based on tdT expression. All lentiviral transductions were performed using a multiplicity of infection of 20 and in the presence of 8 μg/mL of polybrene. Cells were washed three times with Hanks balanced salt solution (HBSS) and collected for *in vitro* evaluation or injection into animals.

### *In vitro* Studies

#### Cell line characterization

All *in vitro* results are from three independent experiments with three replicates of each condition. To evaluate the relationship between cell number and BLI signal, 1×10^4^, 5×10^4^, 1×10^5^, 1.5×10^5^, and 5×10^5^ 4T1BR5-FLuc or 4T1-RLuc cells were seeded in each well of 24-well plates. We acquired fluorescent images of each plate. We then added 10 μL of D-luciferin (30 mg/mL; Syd Labs, Inc., MA, USA) or 10 μL of h-Coelenterazine (150 μg/mL; NanoLight Technology, Prolume, AZ, USA) to the growth medium in each well and BLI images were collected for up to 35 min. All images were acquired using a hybrid optical/X-ray scanner (IVIS Lumina XRMS *In vivo* Imaging System, PerkinElmer). Signal was measured with region-of-interest (ROI) analysis using LivingImage Software (Perkin Elmer). An ROI was drawn around each well to measure the radiant efficiency (p/s/cm^2^/sr/uW/cm^2^) for fluorescence images and average radiance (p/s/mm^2^/sr) for bioluminescence images. The mean signal across replicates was determined for each independent experiment.

#### Cross reactivity

To assess *in vitro* cross reactivity, we seeded two identical 24-well plates with 1 × 10^5^ 4T1-RLuc, 4T1BR5-Fluc, 4T1 naïve cells, and equivalent volume of media. We added 10 µL of d-Luciferin to each well in plate 1 and 10 µL of h-Coelenterazine to each well in plate 2. Images were acquired for up to 35 min and an ROI was drawn around each well to measure the average radiance (p/s/mm^2^/sr). The mean signal across replicates was determined for each independent experiment.

#### Transwell Migration Assay

A FluoroBlok™ Multiwell Insert System was used with an 8um porous polyethylene terephthalate membrane (Corning, Corning NY, USA). We seeded 5×10^4^ cells (4T1-RLuc or 4T1BR5-Fluc) in 75 cm^2^ flasks. At 48 h post seeding, 650 µL of new or conditioned DMEM was collected and used for the bottom chamber of the transwell plate. We then seeded 2.5×10^4^ cells (4T1-RLuc or 4T1BR5-Fluc) in the upper chamber of the transwell insert in 100ul of new DMEM. After 24 h, the membranes were fixed in ethanol for 5 min, washed with PBS, and stained with Hoechst 33342 (10 μg/mL in water) for 5 min. Membranes were cut out with a scalpel and mounted in 90% glycerol onto slides. Three random images were taken of the lower side of each membrane using an Invitrogen EVOS FL Auto Cell Imaging System and the mean fluorescence signal was calculated.

#### CD:UPRT Functionality Testing

To assess the functionality of the CD-UPRT gene *in vitro*, Vybrant MTT assays were used. 2×10^4^ 4T1-CD cells were seeded in each well of 96-well plates and incubated in either the desired concentration of 5'FC (diluted in DMEM) or incubated in DMEM alone. Ten microliters of MTT solution was added to each well and absorbance at 450nm was measured using a microplate spectrophotometer (Fluoroskan Ascent FL, ThermoLabSystems) at 24, 48 and 72 h. This experiment was repeated for 4T1BR5-CD cells.

### *In vivo* Studies

Animals were cared for in accordance with the standards of the Canadian Council on Animal Care, and under an approved protocol of the University of Western Ontario's Council on Animal Care (2015-0558). Six to eight-week-old female nu/nu mice were obtained from Charles River Laboratories (Willington, MA, USA).

#### Contralateral tumor model

Mice received a lower right mammary fat pad (MFP) injection of 300,000 4T1-Rluc or 4T1-CD cells and a lower left MFP injection of 300,000 4T1BR5-Fluc cells on day 0 (Figure 1A; n=5). RLuc BLI was performed on days 0 and 7 and FLuc BLI performed on days 1 and 8. Additional BLI was performed for experiments with CD expressing cells on days 13 (RLuc) and 14 (FLuc). For experiments with CD expressing cells, mice receiving 4T1-Rluc and 4T1-CD cells both received intraperitoneal injections of 5'FC (250 mg/kg/day) on days 7 to 14 (Figure S4A; n=8). Experimental endpoint was considered the final imaging timepoint for each group (day 8 and 16 respectively).

#### Intratumoral model

Mice received a lower right mammary fat pad (MFP) injection of 300,000 4T1BR5-Fluc cells on day 0 and an intratumoral injection of 300,000 4T1-Rluc or 4T1-CD cells on day 7 (Figure S5A; n=8). FLuc BLI was performed on days 0, 4, 8 and 16 and RLuc BLI performed on days 7 and 15. Mice receiving 4T1BR5-Fluc and 4T1BR5-CD cells both received intraperitoneal injections of 5'FC (250 mg/kg/day) on days 8 to 16. Mice were sacrificed at predetermined endpoints based on either excessive weight loss (>15%), the size of the MFP tumor (> 2 cm^3^) and/or the presence of excessive ulceration.

#### Metastatic tumor model

Mice received a lower right MFP injection of 300,000 4T1-Rluc cells. MFP tumors grew for seven days prior to all mice receiving an intracardiac (IC) injection of 2x10^4^ 4T1BR5-FLuc cells in 0.1 mL of HBSS (Figure 2A; n=5). An IC injection was used to try to mimic the natural metastatic spread of cancer cells in the circulation, and to avoid the excessive trapping in the lungs that is seen when these cells are injected via a tail-vein injection. Injections were performed under image guidance using a Vevo 2100 ultrasound system (VisualSonics Inc.). RLuc BLI was performed on days 0, 6, 13 and 19. FLuc BLI was performed on days 7, 14 and 20. For experiments with CD expressing cells, mice receiving 4T1BR5-FLuc and 4T1BR5-CD cells both received intraperitoneal injections of 5'FC (250 mg/kg/day) on days 10 to 20 (Figure 4A; n=11). Mice were sacrificed at predetermined endpoints based on either excessive weight loss (>15%), the size of the MFP tumor (> 2 cm^3^) and/or the presence of excessive ulceration.

#### BLI Procedure

BLI was performed using a hybrid optical/X-ray scanner (IVIS Lumina XRMS *In vivo* Imaging System, PerkinElmer). Mice were anesthetized with isofluorane (2% in 100% oxygen) using a nose cone attached to an activated carbon charcoal filter for passive scavenging. For RLuc BLI, anesthetized mice received a 20 μL intravenous injection of h-Coelenterazine (150 μg/mL) and BLI images were captured for up to 30 min. For FLuc BLI, anesthetized mice received a 100 μL intraperitoneal injection of d-Luciferin (30 mg/mL) and BLI images were captured for up to 35 min.

#### Image Analysis

BLI signal was measured with region-of-interest (ROI) analysis using LivingImage Software (Perkin Elmer). ROIs were drawn throughout the mouse body of RLuc and FLuc image sets for each mouse.

#### Histology

At endpoint, mice were sacrificed by isoflurane overdose and perfused with 4% paraformaldehyde via the left ventricle. Tissues that had suspected lesions based on *in vivo* imaging were removed and cryopreserved in ascending concentrations of sucrose (10, 20, and 30% w/v) for 24 h each, immersed in optimal cutting temperature (OCT) compound, and frozen using liquid nitrogen. Contiguous 10 μm frozen sections were collected and select sections were stained with hematoxylin and eosin (H&E), DAPI, Anti-GFP, Anti-Rluc. Stained sections were imaged using an Invitrogen EVOS FL Auto Cell Imaging System.

#### Statistics

All statistics were calculated using GraphPad Prism 7 Software. Data were expressed as mean ± SEM for *in vitro* and *in vivo* studies and analyzed by Student's t test when comparing two groups with equal variance and by a Mann Whitney test when comparing two groups with unequal variance. An ANOVA was used to compare more than two groups with equal variance and a Kruskal-Wallis test was used to compared more than two groups with unequal variance. Survival times of mouse groups were analyzed using a log-rank test. Differences were considered statistically significant at *p < 0.05, **p < 0.01, ***p < 0.001, and ****p < 0.0001.

## Supplementary Material

Supplementary figures.

## Abbreviations

CTC: circulating tumor cell
CAR-T cell: chimeric antigen receptor T cell
CDUPRT: cytosine deaminase-uracil phosphoribosyltransferase
HSV-TK: herpes simplex virus thymidine kinase
TNF: tumor necrosis factor
PET: positron emission tomography
MFP: mammary fat pad
BLI: bioluminescence imaging
RLuc: *Renilla* luciferase
FLuc: *Firefly* luciferase
GFP: green fluorescent protein
tDT: dTomato
5'FC: 5'-fluorocytosine
5'FUMP: 5' fluoruridine monophosphate
