# Supplementary material for: Engineering Circulating Tumor Cells as Novel Cancer Theranostics
Source: Theranostics. 2020 Jun 29;10(17):7925–37. doi: 10.7150/thno.44259 (PMC7359075; doi:10.7150/thno.44259)
Supplement: Supplementary file 1 — Supplementary figures. [file thnov10p7925s1.pdf]

Supplementary Results

Monitoring Self-Targeted Therapy in a Contralateral Orthotopic Tumor Model

We performed *in vivo* experiments whereby 4T1-RLuc (n=4) or 4T1-CD (n=4) cells were implanted into the right MFP and 4T1BR5-FLuc into the contralateral MFP (Figure S4A). All mice were treated with 5'FC daily from days 8 to 14. As visualized with RLuc BLI, on day 7 before treatment, both 4T1-CD and 4T1-RLuc cells were seen in the contralateral MFP tumor and RLuc signal at this site was not significantly different between mouse cohorts (Figure S4B-C). At day 14 following 5'FC treatment, FLuc BLI signal was observed in both contralateral and ipsilateral MFPs and signal was not significantly different between the two mouse groups in both MFPs (Figure S4E-F). Thus, a treatment effect was not observed in this model. At endpoint, areas of necrosis were evident in MFP tumors from both mouse cohorts using hematoxylin and eosin (H&E) staining (Figure S4G).

Intratumoral Injection of Therapeutic Cancer Cells Can Treat Orthotopic Tumors

We hypothesized the lack of therapeutic effect in the previous experiment may have been due to insufficient numbers of 4T1-CD cells migrating into and proliferating within the contralateral MFP tumor prior to 5'FC administration. Immunostaining of mice sacrificed on day 7 confirmed the presence of both 4T1BR5-FLuc and 4T1-CD cells in the left MFP, but a relatively low ratio of 4T1-CD to 4T1BR5-FLuc cells was noted (Figure S4D). To further test our hypothesis, we allowed the 4T1BR5-FLuc tumors to grow for 7 days prior to injecting $3x10^5$ 4T1-RLuc or 4T1-CD cells intratumorally and treated all mice with 5'FC daily from days 8 to 16 (Figure S5A). 4T1 cells were visualized with RLuc BLI on days 7 and 15, and 4T1BR5 cells with FLuc BLI on days 0, 4, 8 and 16 (Figure S5A). Importantly, RLuc BLI of mice intratumorally injected with 4T1-CD cells was significantly higher on the day prior to 5'FC initiation compared to mice in previous experiment that had the same cells home from the contralateral MFP ($p<0.05$; Figure S5B-C). At day 15 after 5'FC treatment, mice that received 4T1-CD cells intratumorally had a larger percent change in RLuc signal compared to mice that received 4T1-RLuc cells, indicating the ability to mitigate therapeutic cancer

cell growth via suicide switch activation (Figure S5B-D). Furthermore, by day 16, mice that received 4T1-CD cells had significantly less FLuc signal compared to mice that received 4T1-RLuc cells, supporting killing of adjacent cells via the bystander effect ($p<0.05$; Figure S5E-F). Measuring FLuc signal by BLI was complicated by the development of tumor ulcerations in both groups, which partially blocked signal. Therefore, we also assessed treatment response by measuring MFP tumor volumes over time with calipers. Mice that received 4T1-CD cancer cells had significantly smaller tumor volumes than control mice at both days 11 and 17 ($p<0.05$; Figure S5G-H).

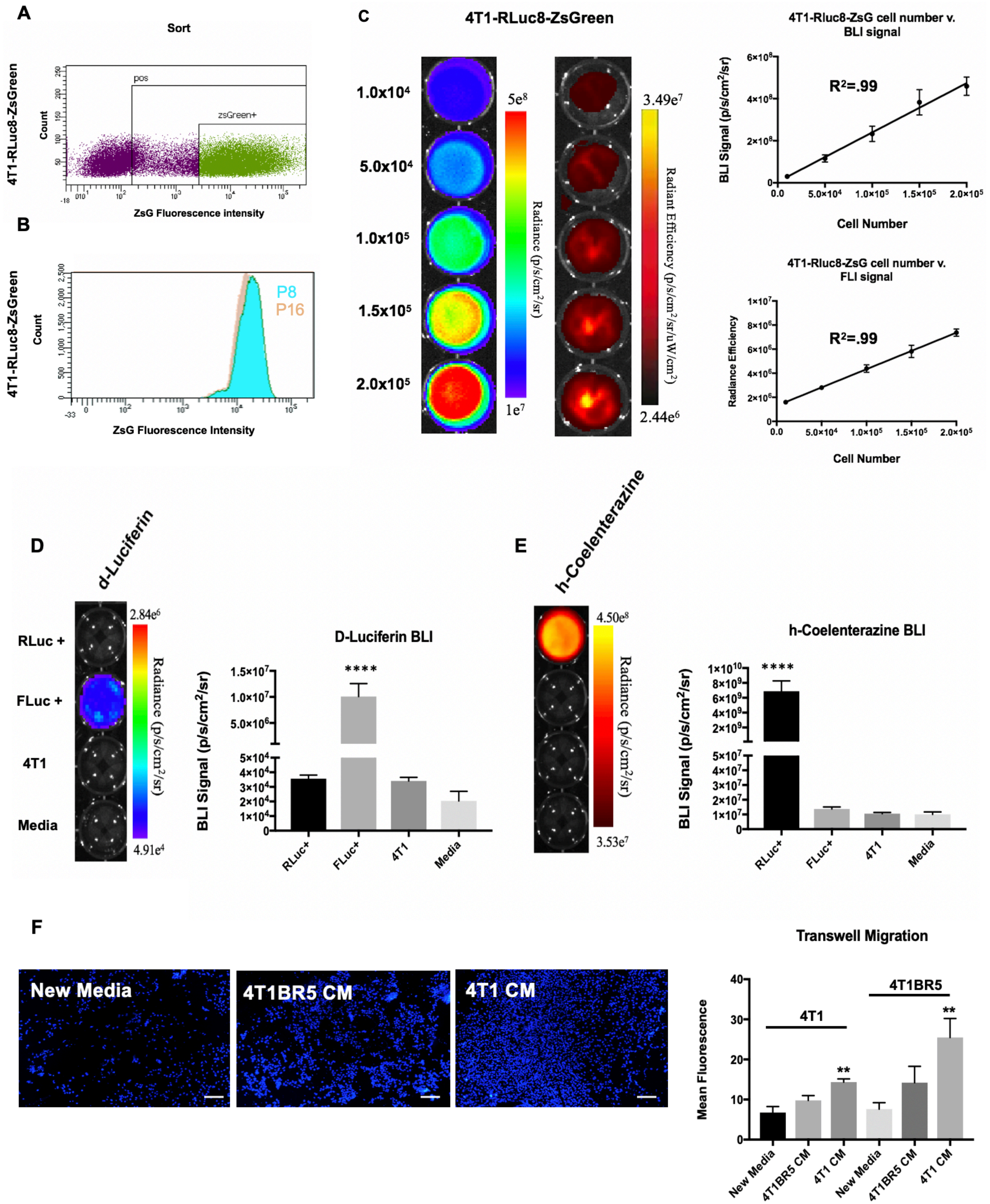
A
Sort
4T1-RLuc8-ZsGreen
Count
pos
zsGreen+
ZsG Fluorescence intensity
B
4T1-RLuc8-ZsGreen
Count
P8
P16
ZsG Fluorescence Intensity
C
4T1-RLuc8-ZsGreen
1.0x10⁴
5.0x10⁴
1.0x10⁵
1.5x10⁵
2.0x10⁵
5e8
1e7
Radiance (p/s/cm²/sr)
3.49e7
2.44e6
Radiant Efficiency (p/s/cm²/sr/uW/cm²)
4T1-Rluc8-ZsG cell number v. BLI signal
R²=.99
BLI Signal (p/s/cm²/sr)
Cell Number
4T1-Rluc8-ZsG cell number v. FLI signal
R²=.99
Radiance Efficiency
Cell Number
D
d-Luciferin
RLuc +
FLuc +
4T1
Media
2.84e6
4.91e4
Radiance (p/s/cm²/sr)
D-Luciferin BLI
BLI Signal (p/s/cm²/sr)
****
RLuc+
FLuc+
4T1
Media
E
h-Coelenterazine
4.50e8
3.53e7
Radiance (p/s/cm²/sr)
h-Coelenterazine BLI
BLI Signal (p/s/cm²/sr)
****
RLuc+
FLuc+
4T1
Media
F
New Media
4T1BR5 CM
4T1 CM
Transwell Migration
Mean Fluorescence
4T1
4T1BR5
**
**
New Media
4T1BR5 CM
4T1 CM
New Media
4T1BR5 CM
4T1 CM

Figure S1: In vitro cell line characterization: 4T1 cells were transduced with a lentiviral vector encoding both RLuc and ZsGreen and sorted to obtain 4T1-RLuc cells (A). No significant change in ZsGreen expression over multiple passages was seen (B). There was a significant positive correlation shown between the number of 4T1-RLuc cells and RLuc/ZsGreen signal (C). 4T1BR5-FLuc cells incubated with D-luciferin demonstrated significantly higher BLI signal than 4T1-RLuc cells, 4T1 parental cells, or equivalent volume of media, and 4T1-RLuc cells did not produce signal significantly different than 4T1 parental cells or media alone (D). Similarly, after the addition of h-coelenterazine, 4T1-RLuc cells had significantly higher signal than 4T1BR5-FLuc cells, 4T1 parental cells, or equivalent volume of media and 4T1BR5-FLuc cells did not produce signal significantly different than 4T1 parental cells or media alone (E). A significant increase in cell migration was seen for 4T1BR5 cells when conditioned media from 4T1 cells was used compared to conditioned media from 4T1BR5 cells or unconditioned media (F). A significant increase in cell migration was also seen for 4T1 cells when conditioned media from 4T1 cells was used compared to unconditioned media (F).

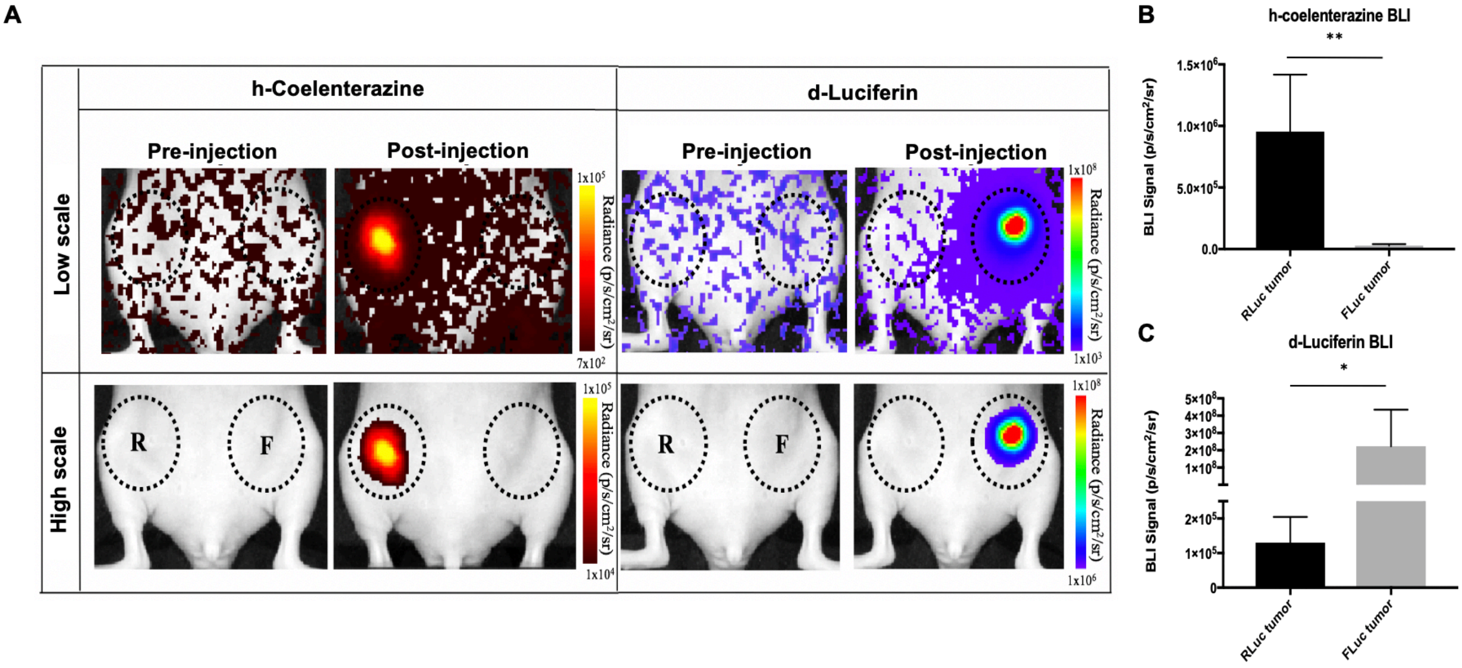

Figure S2: In vivo cross reactivity: 4T1-RLuc cells were implanted into the right MFP of nude mice (n=5) and 4T1BR5-FLuc cells were implanted into the contralateral (left) MFP (A). This allowed us to validate the lack of substrate cross-reactivity *in vivo* at early time points after cell injection. On Day 0, 4T1-RLuc cells only showed signal after administration with h-coelenterazine and signal in the right MFP was significantly higher than the left (B). Similarly, on Day 1, 4T1BR5-FLuc cells only showed signal after administration of d-Luciferin and signal in the left MFP was significantly higher than in the right MFP (C).

A

Belly up days 19/20	Mouse 1	Mouse 2	Mouse 3	Mouse 4	Mouse 5	Mean +/- SEM
Total tumors	11	18	7	7	10	10.6+/- 2.0
RLuc+/ Fluc-	2	1	0	0	0	0.6+/- 0.4
RLuc-/ Fluc+	0	0	0	0	1	0.2 +/- 0.2
Rluc +/Fluc+	9	17	7	7	9	9.8+/-1.86

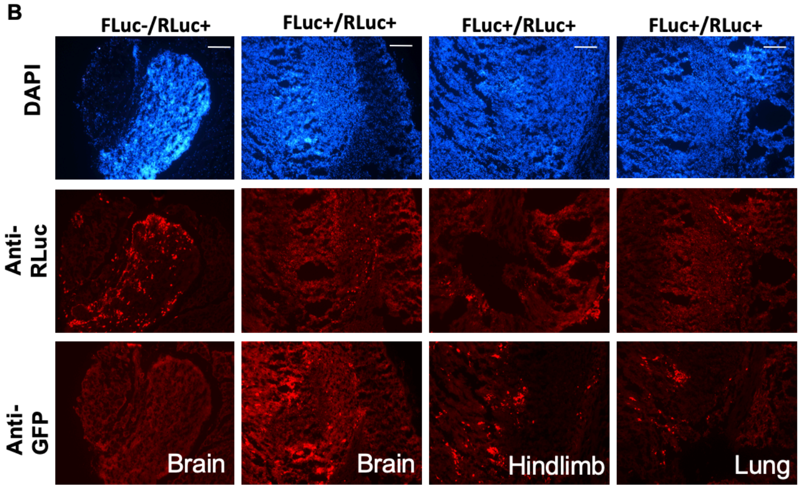

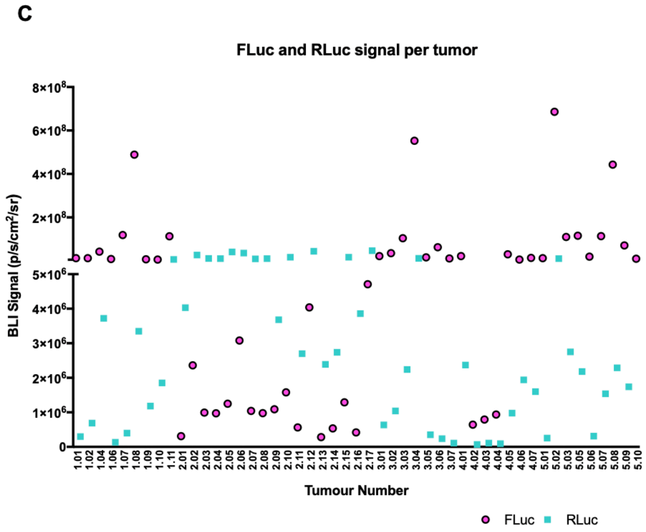

Figure S3: At endpoint, the number of metastases that were composed of both 4T1-RLuc and 4T1BR5-FLuc cells was significantly higher than the number of metastases that were either 4T1-RLuc-positive only or 4T1BR5-FLuc-positive only (A). The presence of both 4T1-RLuc and 4T1BR5-FLuc cells in metastases was confirmed histologically in four biological replicates (scale bar = 500 microns) (B). Using dual-BLI, we detected some whole-body metastases that had stronger FLuc signal than RLuc signal as well as metastases that had stronger RLuc signal than FLuc signal (X-axis values= mouse number followed by tumor number) (C).

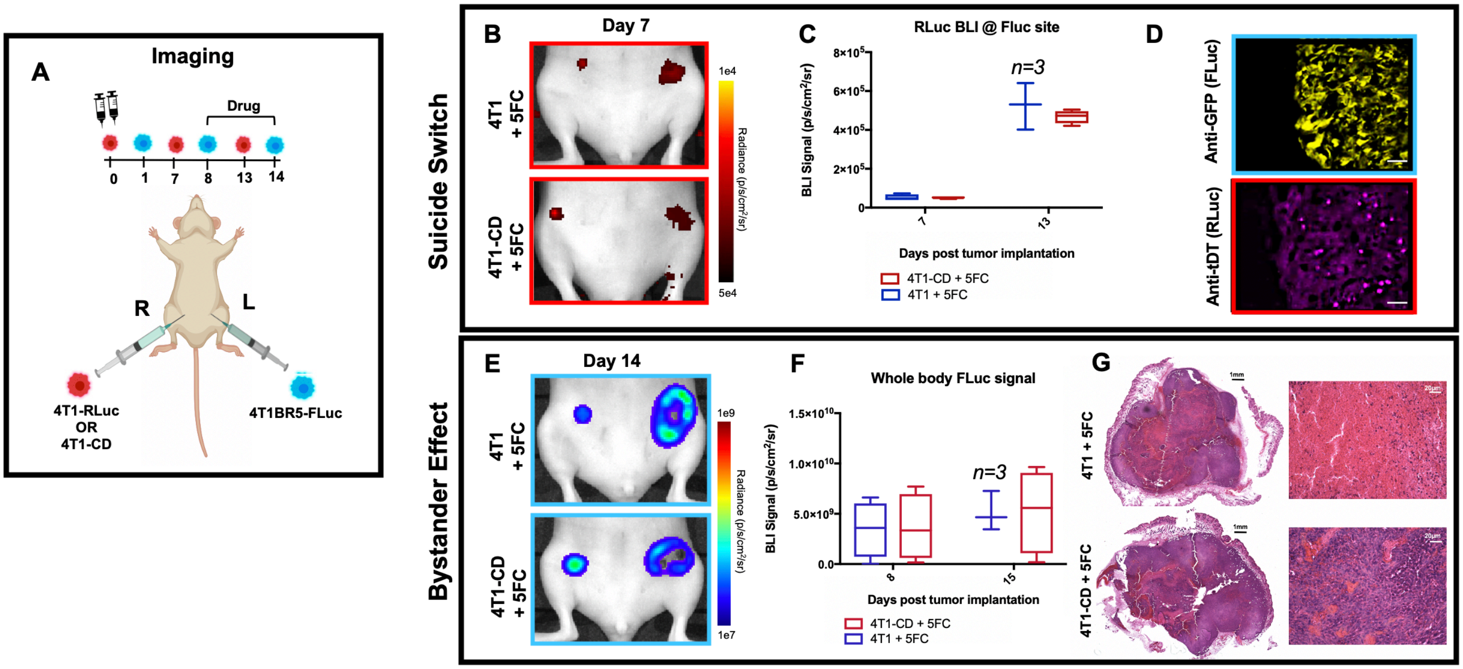

Figure S4: Experimental timeline for contralateral tumor treatment (n=8) (A). All mice were treated with 5'FC daily from days 8 to 14. As visualized with RLuc BLI, on day 7 before treatment, both 4T1-CD and 4T1 cells can be seen in the contralateral MFP tumor and RLuc signal at this site was not significantly different between mouse cohorts (B/C). tDT expressing therapeutic cells were visualized in the contralateral MFP using fluorescence microscopy at day 7 before drug administration (scale bars=200 microns) (D). At day 14 following treatment, FLuc BLI signal was observed in both contralateral and ipsilateral MFPs and signal was not significantly different between the two mouse groups (E/F). At endpoint, areas of necrosis were evident in MFP tumors from both mouse cohorts using hematoxylin and eosin (H&E) staining (black scale bars= 1mm; white scale bars= 20 microns) (G).

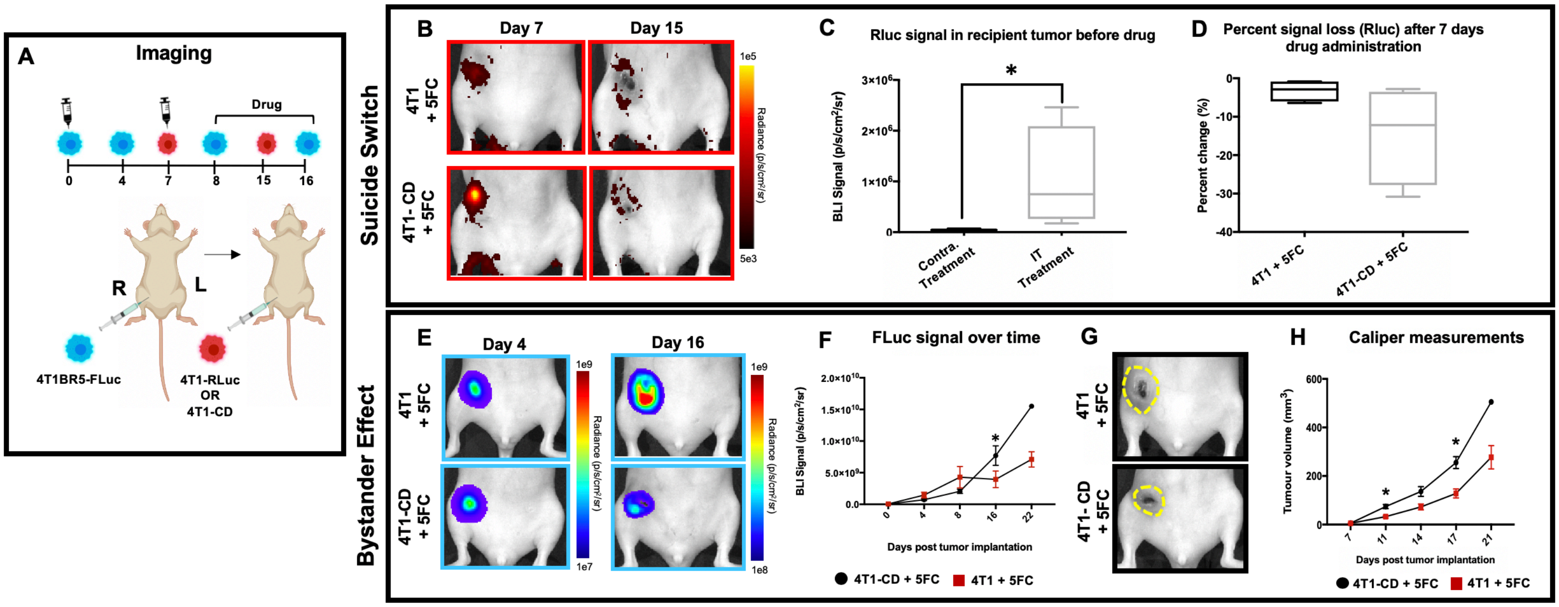

Figure S5: Experimental timeline for intratumoral injection of therapeutic cancer cells (n=8) (A). All mice were treated with 5'FC daily from days 8 to 16. 4T1-Rluc and 4T1-RLuc/CD cells were visualized in the left MFP with BLI on days 7 and 15 (B) RLuc BLI of mice intratumorally injected with 4T1-CD cells was significantly higher than in mice that had the same cells home from the contralateral MFP the day prior to treatment initiation (C). Mice that received 4T1-CD cells intratumorally had a larger percent signal loss of RLuc signal compared to mice that received 4T1-RLuc cells, indicating the ability to mitigate therapeutic cancer cell growth via suicide switch activation (D). By day 16, mice that received 4T1-CD cells had significantly less FLuc signal compared to mice that received 4T1-RLuc cells, indicating kill of adjacent cells via the bystander effect (E/F). Mice that received 4T1-CD cancer cells had significantly smaller tumor volumes than control mice at both days 11 and 17 (H).

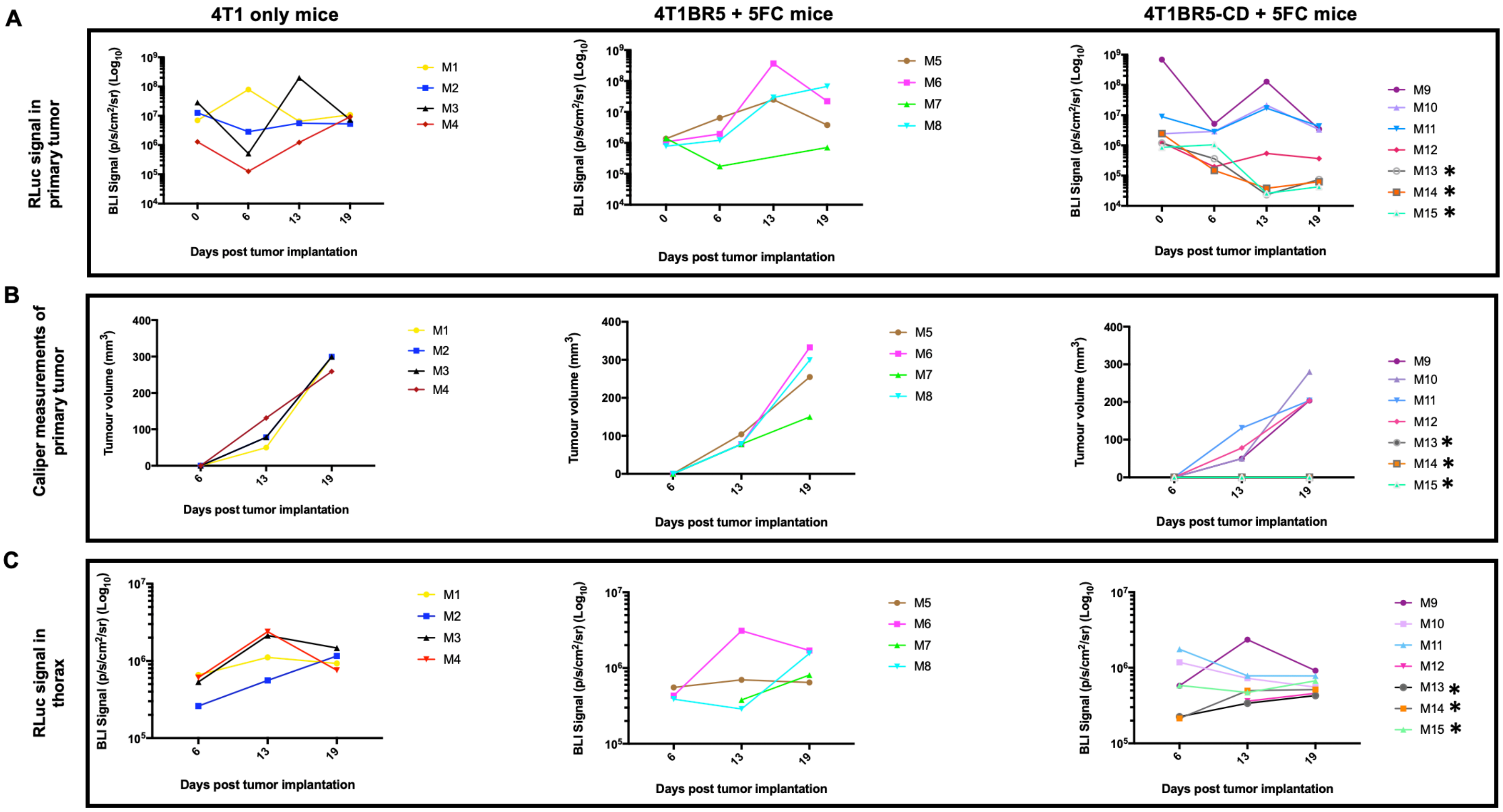

Figure S6: Individual mouse treatment response: RLuc signal the in primary tumor over time (A). Caliper measurements of primary tumor over time (B). RLuc signal in thorax over time (C). Full responders are denoted with an asterisk.
